# Thermosensory thalamus: parallel processing across model organisms

**DOI:** 10.3389/fnins.2023.1210949

**Published:** 2023-10-13

**Authors:** Tobias M. Leva, Clarissa J. Whitmire

**Affiliations:** ^1^Max Delbrück Center for Molecular Medicine in the Helmholtz Association (MDC), Berlin, Germany; ^2^Neuroscience Research Center, Charité-Universitätsmedizin Berlin, Berlin, Germany; ^3^Institut für Biologie, Humboldt-Universität zu Berlin, Berlin, Germany; ^4^Queensland Brain Institute, The University of Queensland, Brisbane, QLD, Australia

**Keywords:** thermosensation, thalamus, thalamocortical, spinothalamic, somatosensory

## Abstract

The thalamus acts as an interface between the periphery and the cortex, with nearly every sensory modality processing information in the thalamocortical circuit. Despite well-established thalamic nuclei for visual, auditory, and tactile modalities, the key thalamic nuclei responsible for innocuous thermosensation remains under debate. Thermosensory information is first transduced by thermoreceptors located in the skin and then processed in the spinal cord. Temperature information is then transmitted to the brain through multiple spinal projection pathways including the spinothalamic tract and the spinoparabrachial tract. While there are fundamental studies of thermal transduction via thermosensitive channels in primary sensory afferents, thermal representation in the spinal projection neurons, and encoding of temperature in the primary cortical targets, comparatively little is known about the intermediate stage of processing in the thalamus. Multiple thalamic nuclei have been implicated in thermal encoding, each with a corresponding cortical target, but without a consensus on the role of each pathway. Here, we review a combination of anatomy, physiology, and behavioral studies across multiple animal models to characterize the thalamic representation of temperature in two proposed thermosensory information streams.

## 1. Introduction

The somatosensory system is a complex network of sensory receptors and neural pathways that enable us to perceive and respond to stimuli from the external environment. Somatosensation refers to the collective experience of these sensory modalities, including touch, temperature, itch, proprioception, and pain. Although touch has been extensively studied, the same cannot be said for temperature, particularly in the context of innocuous stimuli. The ability to perceive changes in temperature is crucial for maintaining homeostasis, detecting potential danger, and responding appropriately to environmental stimuli by serving as necessary information used during active haptic exploration. Hence, investigating the neural mechanisms underlying non-painful thermal stimulation is critical for understanding the broader context of somatosensation. In this article, we focus on the neural pathways implicated in thermosensation and highlight the relevance of two parallel thalamic nuclei and their cortical targets in thermal perception.

Thermal information is transduced by Aδ and C primary afferent fibers innervating the skin that terminate primarily in laminae I and II of the ipsilateral dorsal horn of the spinal cord for sensations arising on the body surface or the spinal trigeminal nucleus for sensations on the face (Light et al., 1979; Light and Perl, 1979a,b; Cervero et al., 1984; Cervero and Connell, 1984; Maxwell and Rethlyi, 1987). Lamina I spinal cord neurons are driven by nociceptive, thermal, and itch stimuli [mouse: (Chisholm et al., 2021); cat: (Craig and Kniffki, 1985; Craig and Hunsley, 1991; Andrew and Craig, 2001; Craig and Dostrovsky, 2001); primate: (Kumazawa and Perl, 1978), rat: (Zhang and Giesler, 2005)]. Spinothalamic projection neurons originating in the superficial laminae of the spinal cord target multiple thalamic nuclei in the mouse (Davidson et al., 2010), the rat (Lund and Webster, 1967; Giesler et al., 1979; Granum, 1986; Ma et al., 1986; Burstein et al., 1990; Iwata et al., 1992; Gauriau and Bernard, 2004a), the cat (Boivie, 1971; Carstens and Trevino, 1978; Jones et al., 1987; Craig et al., 1989; Stevens et al., 1989; Katter et al., 1991; Craig, 2003a; Klop et al., 2005), and the primate (Boivie, 1979; Willis et al., 1979; Hayes and Rustioni, 1980; Apkarian and Hodge, 1989a,b; Gingold et al., 1991; Craig, 2004), in addition to several other non-thalamic targets (Kuner and Kuner, 2021) including the lateral parabrachial nucleus of the brainstem (Light et al., 1993; Bester et al., 2000).

The lateral parabrachial nucleus is responsive to cutaneous thermal inputs (Nakamura, 2011), receives collateral projections from spinothalamic neurons (Hylden et al., 1989; Li et al., 2006; Al-Khater and Todd, 2009), has distinct subregions for cold and warm activated neurons (Nakamura and Morrison, 2008, 2010; Geerling et al., 2016), and projects to the thermoregulatory center of the preoptic area of the hypothalamus (Nakamura and Morrison, 2008, 2010) as well as the central amygdaloid nucleus (Yahiro et al., 2023). Inactivation of lateral parabrachial neurons eliminates autonomic thermoregulatory responses, such as skin cooling-evoked brown fat and shivering thermogenesis and skin warming-evoked cutaneous vasodilation (Kobayashi and Osaka, 2003; Nakamura and Morrison, 2008, 2010). Further, lesions of the lateral parabrachial nucleus (Yahiro et al., 2023), but not the thalamus (Yahiro et al., 2017), can lead to drastic impairments on thermoregulatory behavioral assays. Therefore, while the thalamus is important for sensory perception (Wolff et al., 2021), the spinoparabrachial pathway is considered critical for thermoregulatory behavior independent of the thalamic representation of temperature, and therefore is not the focus of this perspective (for a review of the thermoregulatory pathway, see Nakamura, 2011; Tan and Knight, 2018).

While there are notable differences in the spinothalamic tract across species (Hodge and Apkarian, 1990), the two major thalamic subdivisions targeted by spinothalamic information traveling to cortex are the medial and the lateral thalamus (Craig, 2004). The lamina I—medial thalamus pathway, or the limbic pathway, sends input to the submedial nucleus to the thalamus [multi-species: (Craig and Burton, 1981); rat: (Yoshida et al., 1991; Iwata et al., 1992; Gauriau and Bernard, 2004a), cat:(Craig and Dostrovsky, 1991), primate: (Craig, 2004; Craig and Zhang, 2006)] which then projects to the anterior cingulate cortex (Xue et al., 2022). The lamina I—medial thalamus pathway is associated with pain processing and emotion (Vertes et al., 2015; Bliss et al., 2016), but has not been implicated in innocuous sensing. Rather, there is evidence that the submedial nucleus of the thalamus is exclusively driven by nociceptive stimuli (Dostrovsky and Guilbaud, 1988), and therefore is not the focus of this perspective on innocuous thermal perception (for a review of thalamocortical involvement in pain, see Albe-Fessar et al., 1985; Yen and Lu, 2013; Groh et al., 2017).

The lamina I—lateral thalamus pathway, or the sensorimotor pathway, is associated with nociceptive and thermal perception in rodents (Milenkovic et al., 2014; Ziegler et al., 2023), cats (Craig et al., 2001), and primates (Craig and Blomqvist, 2002). Within the lamina I—lateral thalamus pathway, there are two distinct subdivisions traveling through anterior and posterior somatosensory thalamic nuclei [rodent: (Lund and Webster, 1967; Ma et al., 1986; Zhang and Giesler, 2005), cat: (Boivie, 1971; Mantyh, 1983), non-human primate: (Pearson and Haines, 1980; Apkarian and Hodge, 1989c), human: (Hong et al., 2011)]. Although the anterior and the posterior thalamic nuclei are characterized by distinct thermal representations and cortical targets, the relevance for one pathway over the other for innocuous thermal sensation remains under debate (Craig et al., 1994; Willis et al., 2002; Montes et al., 2005). Here, we propose that the anterior and posterior thalamic thermosensory streams represent two parallel processing pathways for distinct features of thermosensation.

## 2. Anterior thermosensory stream

The anterior thermosensory stream is characterized by the ventral somatosensory thalamic nuclei targeted by spinothalamic neurons. Across species, lamina I spinothalamic tract neurons send axon collaterals to the ventral posterolateral (VPL) and ventral posteromedial (VPM) nuclei as well as to the ventral posterior inferior (VPI) nucleus in primates (Willis et al., 2001). These ventral somatosensory thalamic nuclei are characterized by their robust somatotopic map (Emmers, 1965) and axonal projections to primary (Bernardo and Woolsey, 1987; Shi and Apkarian, 1995) and secondary (Krubitzer and Kaas, 1992) somatosensory cortex for VPL/VPM and VPI, respectively. Therefore, the anterior thermosensory stream is defined as lamina I spinothalamic neurons projecting to ventral somatosensory thalamus and then on to somatosensory cortex.

While traditionally viewed as mechanosensory nuclei, ventral somatosensory thalamus encodes pain (Kenshalo et al., 1980; Casey and Morrow, 1983; Chung et al., 1986; Apkarian and Shi, 1994; Lenz et al., 1994), temperature (Bushnell et al., 1993), and proprioception (Francis et al., 2008). Discriminative mechanosensory information reaches somatosensory ventral thalamus via the dorsal column nuclei (Turecek et al., 2022) while temperature and nociceptive information arrives via the lamina I spinothalamic tract (Hodge and Apkarian, 1990; Willis et al., 2001). The density of the spinothalamic innervation of ventral somatosensory thalamic nuclei is less than that seen for posterior somatosensory thalamus (Al-Khater et al., 2008) which has led to the hypothesis that ventral thalamus is not critical for thermosensation (Craig et al., 1994). However, there is significant evidence that this pathway is also involved in thermosensation beyond mechanosensation (Willis et al., 2002). First, thermally evoked responses have been recorded at each stage of the anterior thermosensory pathway: in VPL-projecting spinothalamic neurons (Chung et al., 1979; Ferrington et al., 1987; Zhang et al., 2006; Davidson et al., 2008), in ventral somatosensory thalamus (Kenshalo et al., 1980; Bushnell et al., 1993; Lenz et al., 1993a; Apkarian and Shi, 1994; Lenz and Dougherty, 1998; Lee et al., 1999), and downstream in primary somatosensory cortex in mice (Milenkovic et al., 2014; Vestergaard et al., 2023) and humans (Guest et al., 2007). Second, human microstimulation studies have elicited cool percepts when the stimulation electrode is located within the ventral caudal thalamus (Lenz et al., 1993b; Ohara and Lenz, 2003). Finally, in monkeys, inactivation of ventral thalamus impaired behavioral performance on both thermosensory and nociceptive tasks, further supporting a role for ventral somatosensory thalamic nuclei in thermal perception (Bushnell et al., 1993). Taken together, this suggests that the anterior thermosensory stream is relevant for thermosensory encoding and perception.

## 3. Posterior thermosensory stream

The posterior thermosensory stream is defined by the posterior somatosensory thalamic nuclei targeted by spinothalamic neurons and their cortical projection target. Identification of this nucleus has required considerable effort across species. In primates, the posterior thalamic nucleus that receives the majority of spinothalamic tract inputs is the posterior part of the ventral medial nucleus, or the VMpo (located posteromedial to the VPL/VPm; previously considered part of PO; see Willis et al., 2002), as identified through both anterograde tracing from superficial spinal cord (Craig, 2004) and retrograde tracing from VMpo (Craig and Zhang, 2006) (for anatomical location of VMPo in humans, see Figure 1 from Montes et al., 2005; for anatomical location of VMPo in non-human primates, from Craig et al., 1994). Lamina I spinothalamic neurons targeting the posterior thalamic nuclei are responsive to thermal stimuli (Gauriau and Bernard, 2004b) and electrophysiology recordings from VMpo in primates have identified thermally responsive neurons (Craig et al., 1994, 1999). Anterograde tracing from VMpo has identified dorsal posterior insular cortex as the primary projection target (Craig, 2014) and posterior insular cortex has been identified as a thermally responsive cortical region in human fMRI studies (Craig et al., 2000). Therefore, the posterior thermosensory stream is defined as Lamina I spinothalamic tract neurons projecting to posterior somatosensory thalamus and then onto posterior insular cortex.

**Figure 1:**
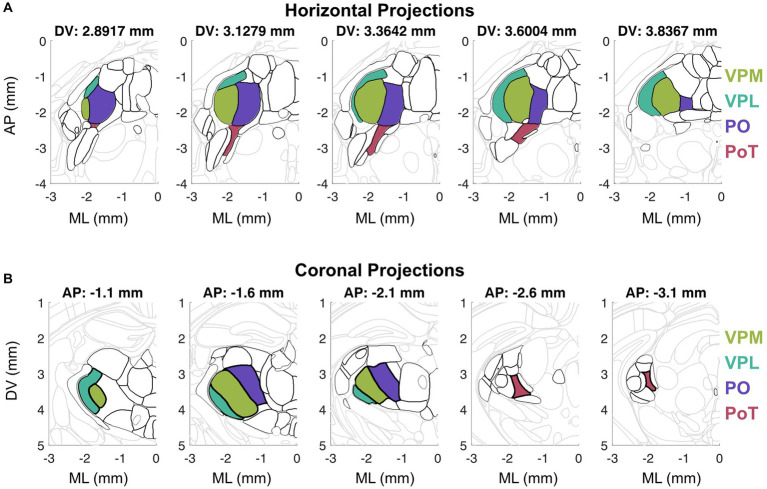
Visualization of horizontal **(A)** and coronal **(B)** sections from the Allen Mouse Brain Common Coordinate Framework (Wang et al., 2020). **(A)** Horizontal sections arranged from dorsal to ventral (left to right) depict thalamic nuclei outlined in black and key thalamic nuclei in color. **(B)** Coronal sections arranged from anterior to posterior (left to right) depict thalamic nuclei outlined in black and key thalamic nuclei in color.

The functional homolog of the primate VMpo has been identified in other species by the dense Lamina I spinal tract innervation, nociceptive and thermosensory response properties, and axonal projections to posterior insular cortex (Craig, 2014). In cats, the ventral aspect of VMb has been described as the VMpo equivalent because this nucleus receives input from lamina I spinothalamic neurons (Craig and Dostrovsky, 2001), is involved in thermosensory behavior (Norrsell and Craig, 1999), and projects to insular cortex (Clasca et al., 1997). In rodents, it was originally believed that the VMpo—insular cortex circuit did not exist (Craig, 2009), but this view has shifted given recent findings depicting a robust thermal representation in mouse posterior insular cortex (pIC) (Vestergaard et al., 2023). In rats, there was initially evidence for VPMpc as the candidate nucleus equivalent to cat VMb (Cliffer et al., 1991; Iwata et al., 1992; Jasmin et al., 2004), but PoT has emerged as the primary candidate (Gauriau and Bernard, 2004a). Similar to VMPo in primates, the PoT nucleus is located posteromedial to the VPL at the caudal end of the PO nucleus (Figure 1). PoT receives the highest proportion of spinothalmic input (Al-Khater et al., 2008), has thermosensitive/nociceptive neurons, and projects to a posterior cortical field we now associate as posterior insular cortex (Gauriau and Bernard, 2004b). Similar to rats, mouse PoT receives spinothalamic input (Davidson et al., 2010) and thalamocortical projections target posterior insular cortex without sending any projections to primary somatosensory cortex (Bokiniec et al., 2023). Therefore, we propose that the rodent functional homolog to VMpo is the PoT nucleus.

**Figure 2:**
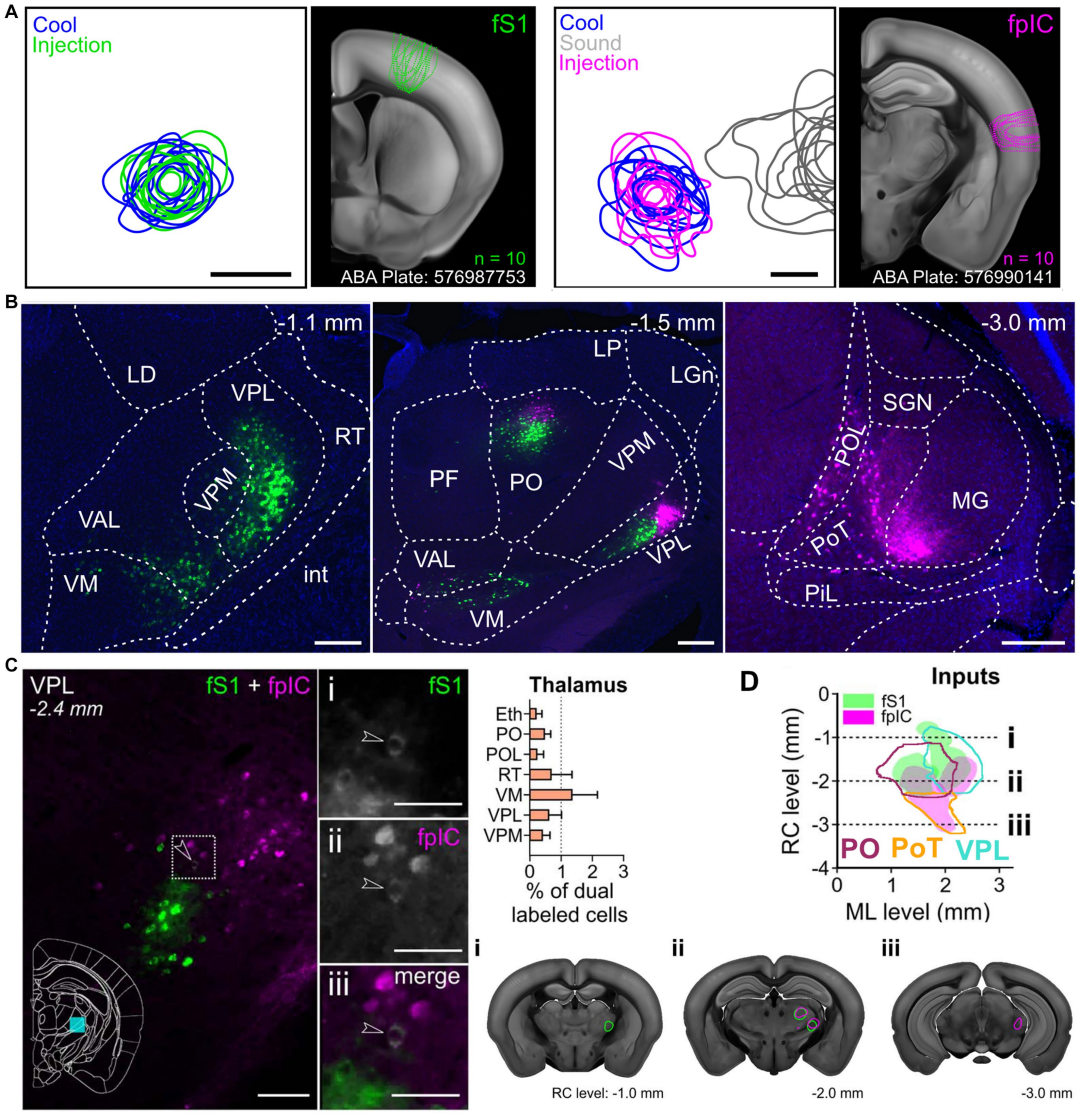
Functionally targeted retrograde tracers injected into forepaw primary somatosensory cortex (fS1) and forepaw posterior insular cortex (pIC) reveal thalamic populations. **(A)** Population injection sites and functional responses of fS1 (*n* = 10 mice). Left plots show 80% contours of the widefield thermal response to cool stimuli (blue) and fluorescence of the tracer (green) (*n* = 10 mice, 5 retrograde and 5 anterograde injections) aligned to peak temperature response in fS1. Image shows outlines of all injection sites localized on a coronal brain slice (ABA Plate: 576987753) from the Allen Brain Atlas. Left plots, Same as for right plots, but for fpIC and including response to 8 kHz sound stimulation (gray). (ABA Plate: 576990141). Scale bars: 500 μm. **(B)** Representative example brain slices of inputs to fS1 (green) or fpIC (magenta) from selected thalamic subregions. The full list of abbreviations is shown in Supplementary Table 1 of Bokiniec et al. (2023). Scale bars: 250 μm. **(C)** Left: Representative micrograph of a coronal brain section showing the VPL nucleus with CTB positive cells projecting to fS1 (i) or fpIC (ii), and one identified cell (iii - white, highlighted by arrowhead) that projects to both fS1 (green) and fpIC (magenta). Right: Percentage of dual labeled cells projecting to fS1 and fpIC in and thalamic nuclei (mean ± SEM, *n* = 5 mice). **(D)** Representation of reconstructed VPL, PO, and PoT inputs to fS1 (green) or fpIC (magenta) (*n* = 5 mice). Left, Plotted as a horizontal view (rostrocaudal vs. mediolateral). Right shows coronal sections highlighting (i) a rostral region with input to fS1 only, (ii) an intermediate rostrocaudal region with input to both fS1 and fpIC, and (iii) a caudal region with inputs to fpIC only. Reproduced from Bokiniec et al. (2023).

The human homolog to VMpo has been identified using cytoarchitecture characteristics of non-human primate VMpo (Blomqvist, 2000). Microstimulation in this region, which is defined as posterior and inferior to the core somatosensory nucleus in human electrophysiology experiments, has evoked thermosensory and nociceptive percepts in humans (Davis et al., 1999). Further, nociceptive-specific units (Davis et al., 1996; Dostrovsky, 2000) as well as cool-responsive units (Davis et al., 1999) have been recorded in this region in humans as well. Localized lesions exist in this thalamic area that is believed to be VMpo (Craig, 2014) in patients experiencing thermanesthesia and thalamic pain (Sprenger et al., 2012). However, it is important to note that this does not appear to be the sole thalamic representation as there are patients with thalamic lesions that do not include VMpo that have still led to abnormal cold sensation and pain (Montes et al., 2005; Kim et al., 2007). There have also been recordings and stimulations in human thalamus that are located much more lateral than VMpo that are cool-responsive (Lenz et al., 1993a; Lenz and Dougherty, 1998), supporting the idea that there are at least two streams for discriminative thermosensory information. Further, retrograde anatomical tracing from thermosensitive cortical regions of the mouse have identified an anterior–posterior distribution in the thalamocortical projection neurons to S1 and pIC, respectively (Figure 2). Therefore, the posterior thermosensory stream is a key pathway for thermal encoding, but not the exclusive pathway for thermosensation.

## 4. The encoding of innocuous temperature in the anterior and posterior thermosensory streams

The existence of two parallel streams of thermosensory information suggests distinct roles of each thalamic nucleus in thermal encoding. In the context of temperature, the putative function of distinct representations have been considered in the frameworks of the sensory and the hedonic aspect of the stimulus (Sewards and Sewards, 2002) as well as exteroception and interoception (Craig, 2002). Here, we first consider the distinctions in the sensory representation itself and then expand on the proposed functional roles of these two parallel streams. While there are multiple stimulus dimensions that could differ, the most salient appears to be the representation of warm and cool in each pathway.

### 4.1. Functional encoding of warm and cool

Upon discovery of discrete cold and warm spots in the skin (Blix, 1882), cool and warm encoding have been explored almost as two separate entities with the majority of studies reporting on the representation of cool stimuli. Historically, electrophysiological recordings investigating cooling sensation were targeting the ventral basal complex (VB), or the anterior stream as we describe here. Cool-sensitive neurons were identified within VB in response to cooling of the tongue (Poulos and Benjamin, 1968; Auen et al., 1980) or limb (Burton et al., 1970; Schingnitz and Werner, 1980). In human studies, cool-evoked neural responses were also identified in ventral somatosensory thalamus (Lenz and Dougherty, 1998; Lee et al., 1999). Cool-evoked thalamic responses showed either transient or sustained response dynamics (Burton et al., 1970). Some VB neurons showed high sensitivity to cool with step-like tuning functions, while others had a more graded response across increasing stimulus intensities (Sakata et al., 1989). Responses in VB to warm stimulation of the limb or tongue were incredibly rare, with some studies reporting no warming cells (Poulos and Benjamin, 1968). Responses to warming have also been uncommon relative to cooling at the spinal cord (Kenshalo et al., 1979; Ferrington et al., 1987; Dado et al., 1994). The notable exception to this lack of warm stimulus encoding in the thalamus was found in response to scrotal stimulation (Hellon and Misra, 1973; Jahns, 1975; Schingnitz and Werner, 1980). While warming the scrotum could elicit a strong evoked response in VB, this response required cortical involvement (Hellon and Taylor, 1982) and was later described as a non-specific activation (Kanosue et al., 1985). Therefore, warm-evoked sensory responses in VB remain elusive. Downstream, in somatosensory cortex, there is a similar lack of warm representation. In human EEG, cooling the glabrous skin of the hand elicited an evoked response over somatosensory cortex, while no evoked potential of any form was measured following rapid warming of the hand (Duclaux et al., 1974). This is consistent with recent imaging studies from somatosensory cortex in mice showing a robust cool response (Milenkovic et al., 2014), but a minimal warm response (Vestergaard et al., 2023).

However, the absence of warm encoding in the anterior thermosensory stream sets up an obvious concern – why is warm not represented here? At the periphery, cool-responsive neurons greatly outnumber the warm-responsive neurons (McGlone and Reilly, 2010). Therefore, the sparse encoding of warm in the anterior thermosensory stream could simply be predicted from the distribution of peripheral receptors and would suggest that warm is not well represented in whole organisms. However, warm is encoded robustly in the hypothalamus, independent of the thalamocortical circuit (Nakamura, 2011; Tan et al., 2016), demonstrating that warm can be represented in the central nervous system. While behavioral sensitivity to warm is lower than to cold (Paricio-Montesinos et al., 2020), humans are fully capable of detecting both transient warm and cool stimulation (Stevens and Choo, 1998) suggesting a discriminative representation of warm must exist in the somatosensory pathway. Alternatively, it is also possible that the cold representation seen in the anterior thermosensory stream is mediated exclusively by slowly adapting mechanoreceptors with thermal sensitivity (Burton et al., 1972) via the dorsal column nuclei (Abraira and Ginty, 2013). While this could underlie some of the thermotactile-responsive neurons in the anterior thermosensory stream, it would not account for the cool-selective units that have been recorded throughout the nucleus. Another possibility is that warm encoding in the anterior stream could be mediated via suppression rather than excitation. Warming stimulation can elicit suppressed responses in cool-responsive primary sensory afferents (Burton et al., 1970; Paricio-Montesinos et al., 2020). Warm-evoked suppression could be difficult to identify in anesthetized recordings because the spontaneous firing rate is artificially low. This must be investigated further to identify whether a robust warm-evoked suppression exists in the anterior thermosensory stream. Overall, the evidence suggests that the anterior stream is primarily involved in cool encoding with minimal impact on the discriminative encoding of warm.

The investigation of the posterior thermosensory stream is far more recent, which has resulted in even fewer investigations of thermosensation in posterior thalamus than ventral thalamus. However, there are electrophysiological recordings that have measured innocuous thermosensory responses in posterior thalamus (Craig et al., 1994) in addition to other modalities such as touch, itch, and pain (Lipshetz et al., 2018), further confirming its involvement in somatosensory processing. Spinothalamic Lamina I terminations in macaque VMPo display a coherent topographic map as assessed using anterograde (Craig, 2004) and retrograde (Craig and Zhang, 2006) tracing techniques suggesting this thalamic region has somatotopy. Additionally, anterograde tracing from the VMPo has also suggested a somatotopic map in the thalamocortical projections (Craig, 2014) to cortex. Microstimulation studies in humans found that while the cool percepts could be evoked in both the core region of somatosensory thalamus (anterior stream) and the posterior region of somatosensory thalamus (posterior stream), warm percepts were evoked more frequently in the posterior region (Ohara and Lenz, 2003). Microstimulation in insular cortex has also elicited non-painful warming percept in humans (Ostrowsky et al., 2000) and recordings in posterior insular cortex of mice have also shown a robust warm representation (Vestergaard et al., 2023). While further research is needed to better understand the neural mechanisms underlying the processing of warm stimuli and its representation in the brain, this suggests that the posterior stream is involved in both cool and warm thermal representation.

### 4.2. Proposed roles for the anterior and posterior thermosensory streams

Parallel pathways for processing sensory information are the rule rather than the exception. In the visual cortical pathway, the ventral stream is believed to underlie visual discrimination while the dorsal pathway is associated with spatial vision and visually guided behaviors (Ungerleider and Mishkin, 1982). The ventral and dorsal visual streams are colloquially known as the “what” and “where” pathways for vision. Similarly, in the auditory circuits, the ventral pathway is associated with sound recognition while the dorsal pathway is associated with sound localization and speech production (Wang et al., 2008). As with vision, these pathways are also referred to as the “what” and “where” pathways for audition.

While these parallel cortical pathways are not considered entirely independent, the identification of two pathways that underlie sensory discrimination and action guidance has led to a shift in the perspective for visual and auditory processing. A similar search for guiding principles in somatosensory processing is underway. Here, we outline the major proposed roles of the distinct thermosensory pathways in somatosensation, with a particular focus on thermosensation.

#### 4.2.1. Sensory discrimination and sensory localization

While these auditory and visual pathways are cortico-cortical circuits that likely vary critically from thalamocortical pathways, it is tempting to try to draw direct parallels between the visual and auditory themes of sensing-for-discrimination and sensing-for-action in the “what” and “where” cortical pathways, respectively, to the proposed thermosensory information streams.

Posterior insular cortex, or the cortical target of the posterior thermosensory pathway, encodes a thermal representation of both warm and cool stimuli (Vestergaard et al., 2023) that is correlated with the perceived temperature rather than the valence of the thermosensation (Craig et al., 2000). This representation of temperature would lead to a more discriminable characteristic (Craig, 2011) than that seen for somatosensory cortex where a warm representation is largely absent (Vestergaard et al., 2023) and therefore could be compatible with a “what” stream of information for thermal encoding (Dijkerman and De Haan, 2007).

In contrast, the somatosensory cortex, or the cortical target of the anterior thermosensory pathway, is highly interconnected with regions involved in sensorimotor processing including motor cortex, and contains a precise somatotopic map of the body surface (Krubitzer and Kaas, 1990). A thermal stimulus is typically provided in concert with a tactile stimulus when interacting with objects or surfaces. Therefore, the more restricted thermal representation here combined with the robust activation of somatosensory cortex by the thermotactile stimulus could provide spatial localization to be primarily associated with the “where” pathway.

Similar to the visual and auditory pathways, these parallel pathways would not contain completely independent information. For example, cool information would be discriminable in the “where” pathway (anterior stream) and spatial information could be decoded from the somatotopic maps of temperature in posterior insular cortex (Vestergaard et al., 2023) in the “what” pathway (posterior stream). Yet the same hypothesis would not be drawn for tactile information. Somatosensory cortex is considered requisite for discriminative tactile information and spatial localization, making it critical for both the “what” and the “where” touch pathway (Diamond et al., 2008). However, as discussed below, it is entirely possible that touch and temperature should not necessarily be considered comparable sensations with identical neural pathways.

#### 4.2.2. Interoception and exteroception

As described thus far, somatosensation is the compilation of multiple submodalities including touch, itch, proprioception, temperature, and pain that sense exteroceptive stimuli, or stimuli that are external to the body. However, it has been proposed that temperature and pain have been categorically misclassified as exteroceptive instead of interoceptive, or relating to stimuli that are internal to the body (Craig, 2002). In this view, interoceptive explicitly includes both visceral sensation and homeostatic sensory capacity (Ceunen et al., 2016). Therefore, the posterior thermosensory stream could be responsible for the interoceptive representation of the body state (Craig, 2003b) including thermosensory encoding.

As such, thermosensation is described as a homeostatic emotion because the valence of the thermal stimulus is dependent on the homeostatic state (Craig, 2007). Practically speaking, this would be equivalent to the pleasant feeling of a cool breeze on a warm sunny day compared to the unpleasant feeling of the same cool breeze during a frigid winter day. Therefore, the thermal representation in the posterior insular cortex, or the cortical target of the posterior thermosensory pathway, is referred to as both the primary thermosensory cortex and the limbic sensory cortex. This is because the posterior insular cortex is highly interconnected with regions involved in valence and homeostasis including the amygdala, hypothalamus, orbitofrontal cortex, and parabrachial nucleus (Yasui et al., 1991; Gehrlach et al., 2020; Bokiniec et al., 2023). In a positron emission tomography (PET) study, orbitofrontal cortex and anterior insular cortex thermal-evoked responses were correlated with subjective thermal experience (i.e., valence) while posterior insular cortex was correlated with objective thermal stimulation (i.e., applied temperature) (Craig et al., 2000). Therefore, it has been proposed that the sensory representation in posterior insular cortex is the limbic sensory substrate for subjective/homeostatic feelings and emotions (Craig, 2002).

Importantly, this view essentially excludes the role of the anterior thermosensory stream in thermal encoding and highlights posterior insular cortex as the key structure for interoception. While posterior insular cortex is tightly interconnected with limbic sensory structures and likely is involved in valence encoding (Gogolla, 2017), it is not necessarily required for homeostatic thermoregulation. As described above, the spinoparabrachial tract is crucial for thermoregulation (Yahiro et al., 2017), independent of the thalamic representation which presumably drives the cortical representation in the posterior insular. This would suggest that posterior insular cortex could constitute the primary discriminative thermal representation, but does not clearly delineate whether thermosensation should be classified as exclusively interoceptive. In comparison, tactile encoding would be considered both interoceptive and exteroceptive. Interoceptive tactile signaling could be stomach distension when feeling ‘full’ after eating while exteroceptive tactile signaling could be the deformation of the skin when grasping an object. Exteroception is inherently important for somatosensation as it would include direct interactions with the external environment. The importance of particular regions of the body, such as the hand and mouth, for exteroception can be visualized in the innervation density of the primary sensory afferents in the skin, as measured anatomically and by perceptual acuity. Both mechanoreceptors and thermally responsive fibers densely innervate skin used for actively sensing the environment, such as the hands or the lips, relative to other body segments that may provide more critical measures of homeostatic state or body temperature, such as the abdomen (Stevens and Choo, 1998; Corniani and Saal, 2020). Taken together, we would propose that thermosensation is involved in both interoception and exteroception and that it is unique from touch in that it has a clear autonomic function in thermal homeostasis.

#### 4.2.3. Valence and discriminative stimulus attributes

The definition of somatosensory encoding has also been considered in the framework of discriminative sensory features and hedonic, or valence, sensory features. While this view has been discussed in the context of pain, and particularly whether pain should be treated as a separate submodality of somatosensation or as a negative valence signal associated with other modalities such as touch, it has parallels to temperature sensing (Sewards and Sewards, 2002). Similar to the natural valence of thermal encoding described above, tactile encoding has a similar separation between sensory discrimination and sensory valence. For touch, a specific class of mechanosensitive fibers has been proposed to mediate affective, or rewarding touch properties, as opposed to sensory discriminative properties (McGlone et al., 2014). Within thermal encoding, human subjects can differentiate between thermal stimuli regardless of homeostatic body temperature while unpleasantness varied with body temperatures, suggesting that valence and sensory features could be represented by the activity of two neuronal populations (Mower, 1976). It has been proposed that the hedonic and discriminative sensory representations can both exist within the ventrobasal and posterior complex of the somatosensory thalamus, but the cortical encoding in primates contains only sensory representations in somatosensory cortex and has both sensory and hedonic representations in the insular cortex (Sewards and Sewards, 2002). In this view, the anterior thermosensory stream would be primarily driven by discriminative sensory features, while the posterior thermosensory stream would be primarily driven by the valence of the thermal stimulation.

However, the anterior thermosensory stream does not contain the full thermosensory representation and therefore would not be the ideal candidate region for thermal discriminative features. Further, we would actually delineate valence into two subcategories. The first classification of valence would be attributed only to the stimulus qualities. In touch, for example, this could be defined as a positive valence associated with a gentle stroke using silk compared to the negative valence associated with a hard stroke using sandpaper. The second classification of valence would be attributed to the integration of the stimulus qualities with the internal state. In touch, this could be described as the positive emotional response generated when tickled while happy compared to the negative response generated when tickled while crying. In this consideration, the first classification of valence based on stimulus properties could exist within each pathway. However, aforementioned human studies found that activity in posterior insular cortex does not correlate with valence, but only with stimulus intensity (Craig et al., 2000), suggesting that the posterior thermosensory pathway is not necessarily representing the first classification of valence. However, consistent with the theory of interoception (Craig, 2002), the second classification of valence would likely be developed in downstream structures from the posterior thermosensory stream. The combination of the reduced sensory representation in the anterior pathway and the evidence against valence representation in either pathway suggests there is insufficient evidence to predict that thermal encoding in these two thermosensory pathways is split well into these two categories of valence and discriminative features.

#### 4.2.4. Thermotactile sensing and temperature discrimination

**Figure 3:**
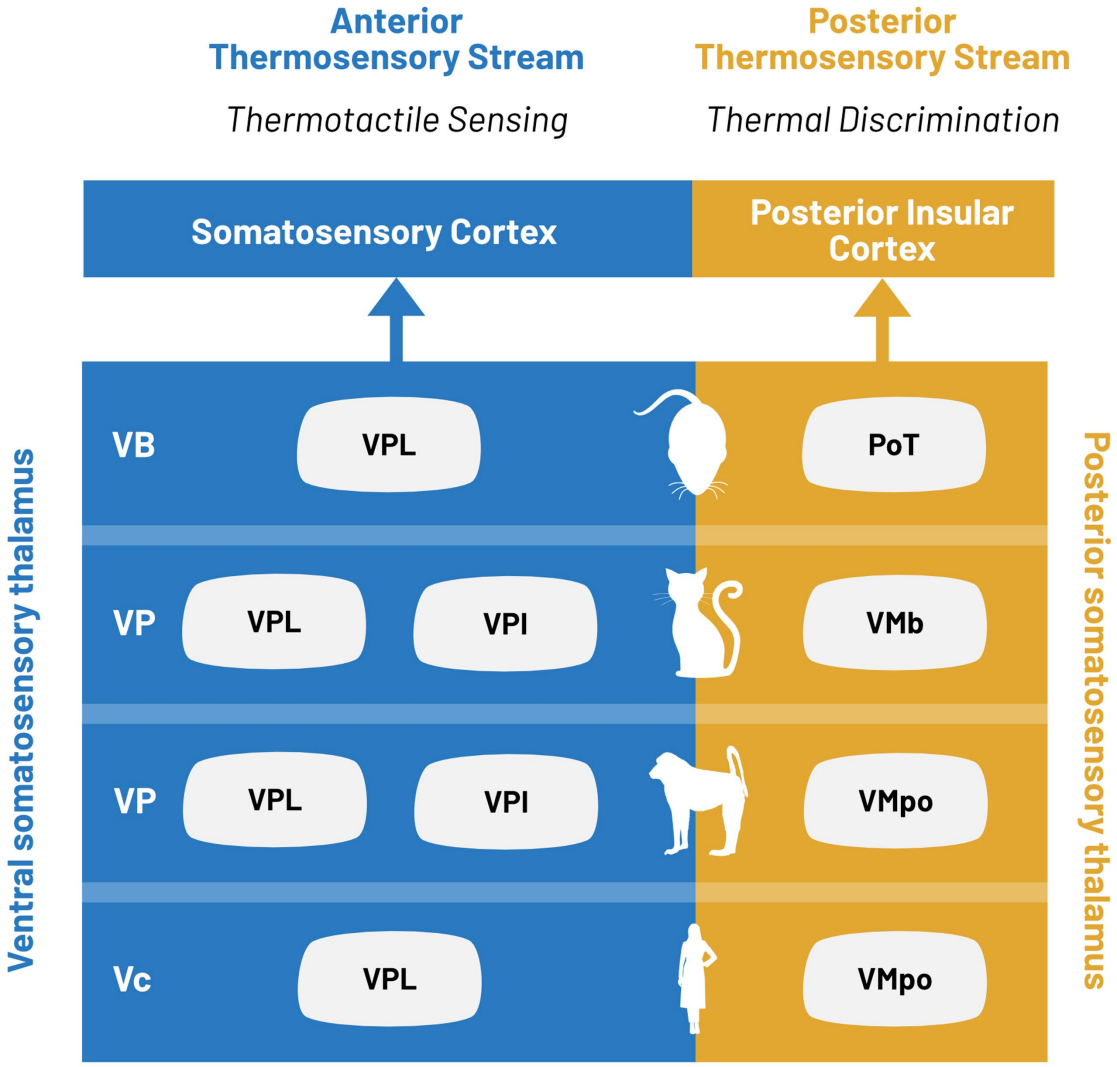
Parallel processing in ventral and posterior thermosensory streams. Thermal information is encoded in two distinct thalamocortical pathways defined as the ventral (blue) and posterior (yellow) thermosensory streams. Thalamic nuclei associated with each stream for limb stimulation are indicated for, from bottom to top: humans, non-human primates, cats, and rodents.

Instead, we propose a synthesis of these theories to suggest that thermal pathways are actually characterized by two distinct functional roles, both of which impact thermosensation. These two functional roles are thermotactile sensing and isolated temperature sensation (Figure 3). Somatosensory cortex has been implicated as a key structure in active sensing, or haptic exploration. The anterior thermosensory pathway has a restricted thermal encoding paired with robust tactile encoding. We propose that the thermal representation in this pathway is primarily involved in thermotactile behaviors. Given that mammalian species are homeotherms, the environmental temperature is usually lower than that of the body. Therefore, the statistics of the natural thermosensory environment are heavily cold biased, which could explain the limited sensory representation in the anterior thermosensory stream.

In contrast, the posterior thermosensory pathway contains the relevant information for both warm and cool encoding to have discriminative thermosensory capabilities. In the cortical target of this pathway, touch and temperature are represented in distinct subpopulations (Vestergaard et al., 2023) which would further enable precise thermosensory encoding. All evidence suggests that the posterior thermosensory pathway is crucial for temperature discrimination, but extensive studies into the thalamic nucleus responsible for this pathway will be required to elucidate its classification within the thalamic hierarchy. This would suggest that posterior thalamus and posterior insular cortex could encode both interoceptive and exteroceptive thermosensory information while providing discriminative thermal information to both the anterior thermosensory stream and the limbic thermosensory pathway. Taken together, we believe this framework could provide a functional segregation between the pathways while remaining consistent with prior proposed models.

## 5. Discussion

The relevant pathways for thermosensory processing in the central nervous system remain an area of active debate. Here we propose a synthesis of research across model organisms to describe two pathways for innocuous thermal perception: the anterior and the posterior thermosensory streams. The anterior thermosensory pathway transmits thermosensory information from the superficial laminae of the spinal cord to the ventral nuclei of the lateral thalamus and ultimately somatosensory cortex. The posterior thermosensory pathway transmits thermosensory information from the superficial laminae of the spinal cord to the posterior nuclei of the lateral thalamus and ultimately posterior insular cortex. While there is an extensive body of work on somatosensory encoding in the ventral somatosensory thalamic nuclei, there is a paucity of data in the posterior somatosensory thalamic nuclei. Anatomical tracing has shown that the thalamic neurons of the anterior and posterior streams are completely non-overlapping (Bokiniec et al., 2023), suggesting parallel information processing in the thalamocortical circuit, but comparative studies investigating thermal encoding properties across these thermal pathways will be required to support, or disclaim, the hypothetical frameworks presented here. While it has been proposed that the anterior and posterior thermosensory streams are designated for sensory-discriminative functions (Vestergaard et al., 2023), interoception (Craig, 2002), or hedonic identity (Sewards and Sewards, 2002), the one organizing principle that remains evident is that temperature is not touch. It is a distinct modality that requires further exploration and future work is required to carefully disentangle the role of these two thalamic pathways on thermal encoding.

## Author contributions

TL and CW contributed to conception and design of the article, developing the literature review, and building a cohesive framework. CW wrote initial manuscript draft. All authors contributed to the article and approved the submitted version.

## Abbreviations

Vc: Ventral caudal
VPL: Ventral posterolateral nucleus
VP: Ventral posterior nucleus
VPI: Ventral margin of the ventral tier nuclei
VB: Ventrobasal complex
PoT: Posterior triangular nucleus
VMb: Basal part of the ventral medial nucleus
VMpo: Posterior part of the ventral medial nucleus
S1: Primary somatosensory cortex
pIC: Posterior insular cortex
